# Upcycling pine-bark into powerful adsorbents: tetracycline removal from aquaculture effluents combining biochar and advanced oxidation processes

**DOI:** 10.1007/s11356-025-37382-4

**Published:** 2026-01-17

**Authors:** Samuel Moles, Rosa Mosteo, Francisca Romero-Sarria, Patricia García-Muñoz, Jorge Rodríguez-Chueca

**Affiliations:** 1 Instituto de Investigación en Ciencias Ambientales de Aragón (IUCA), Universidad de Zaragoza, C. de Pedro Cerbuna, 12, 50009 Saragossa, Spain; 2 Department of Industrial Chemical and Environmental Engineering, Escuela Técnica Superior de Ingenieros Industriales, Universidad Politécnica de Madrid, C/José Gutiérrez Abascal 2, 28006 Madrid, Spain; 3 Department of Inorganic Chemistry and Institute of Materials Science, Joint Center University of Seville-CSIC, Av. Américo Vespucio, 41092 Seville, Spain; 4 Departament of General Chemistry, Escuela Universitaria Politécnica La Almunia Universidad de Zaragoza, C. Mayor, 5, 50100, La Almunia de Doña Godina, Saragossa, Spain

**Keywords:** Biochar, Aquaculture, Wastewater, Antibiotic removal, Adsorption

## Abstract

The presence of antibiotics in aquaculture wastewater poses environmental and public-health risks by disrupting aquatic ecosystems and promoting the spread of antibiotic-resistant bacteria. This study evaluates pine-bark biochars activated under different atmospheres for the removal of tetracycline from real aquaculture wastewater and examines their combined use with peroxymonosulfate as an oxidant. The biochars were produced by pyrolysis and activated using carbon dioxide or humid argon. Carbon-dioxide activation generated a larger surface area and a more developed porous structure than humid-argon activation, which resulted in higher adsorption performance. Batch experiments achieved 80–100% tetracycline removal in real aquaculture wastewater containing competing ions and dissolved organic matter. Adsorption kinetics followed the pseudo-second-order model, indicating that chemisorption governed the process, while intraparticle diffusion contributed but was not the controlling step. The solution pH strongly influenced adsorption, with maximum removal under alkaline conditions. Results suggest that aromatic ring interactions, hydrogen bonding and surface complexation were predominant adsorption mechanisms. Combining biochar with peroxymonosulfate enhanced tetracycline removal through a synergistic effect, reaching up to 99% with very low oxidant dosages. These findings highlight pine-bark biochar as a promising and sustainable metal-free material for treating contaminants of emerging concern in aquaculture wastewater.

**Graphical Abstract:**

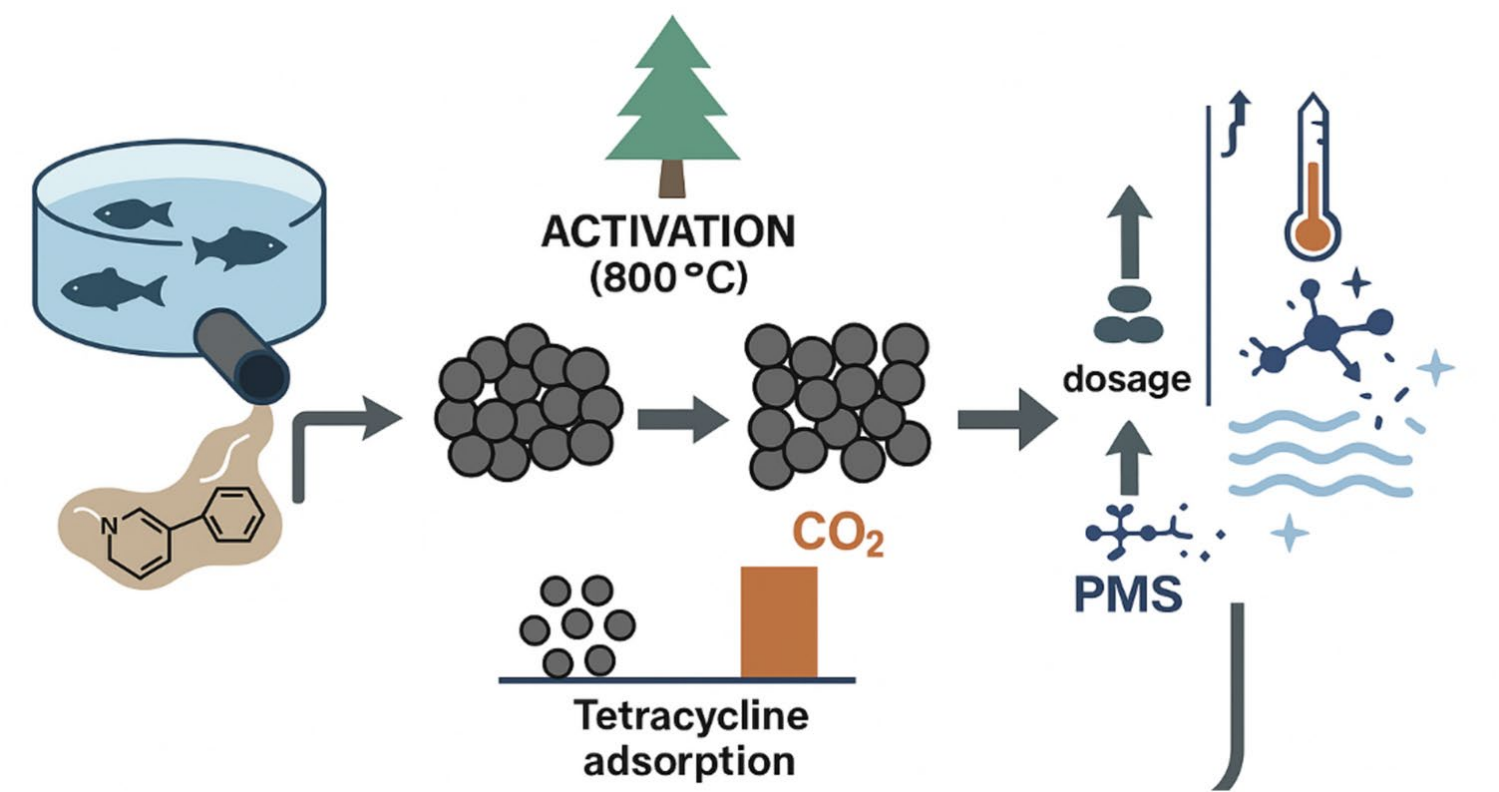

## Introduction

Aquaculture refers to the cultivation of fish, shellfish, mollusks, and other aquatic organisms. The contamination of aquatic ecosystems by antibiotics, particularly in aquaculture is an issue of special concern. The misuse of antibiotics, for instance to prevent bacterial infections or promote fish growth, leads to the introduction of large quantities of antibiotics directly into the aquaculture system. Two of the most frequently used antibiotics in aquaculture are tetracycline and amoxicillin, which have a wide range of action against different types of bacteria and are therefore widely applied (Kim et al. 2013). This practice allows these compounds to leach into nearby water bodies, resulting in detectable levels of antibiotics in both wastewater and surface waters. The widespread occurrence of antibiotics in aquaculture wastewater underscores the need for more sustainable practices in the industry.

Furthermore, conventional wastewater treatment plants (WWTPs) are not designed to efficiently remove pharmaceuticals and personal care products (PPCPs), such as antibiotics (Reis et al. 2020). Several studies have indicated that these processes, e.g. coagulation, sedimentation and biological treatment are not successful in total removal of antibiotics (Gothwal and Shashidhar 2015). As a result, the incorporation of antibiotics into the water cycle promotes the spread of antibiotic resistance genes. Consequently, in the last decades, various physical and chemical approaches have been studied for the removal of antibiotics from water. For instance, membrane filtration processes, advanced oxidation processes (AOPs) and adsorption are some of the state-of-the-art treatment technologies investigated to assess their capabilities in antibiotic removal from wastewater (Miklos et al. 2018). While both membrane filtration and AOPs have sometimes shown a lack of selectivity, sustainability and effectiveness in real scenarios, activated carbon adsorption is a well-stablished process applied at industrial scale because it is highly effective at removing organic pollutants depending on its structural characteristics (Rodríguez-Chueca et al. 2023).

Biochar is a carbon-rich material produced by heating organic biomass (such as wood, crop residues, or manure) at high temperatures (typically 300–700 °C) in a low-oxygen environment, a process known as pyrolysis or gasification (Luo et al. 2022; Feng et al. 2020; Yang et al. 2022). The physicochemical characteristics of this solid depend on the material used as feedstock, as well as on the operational conditions of the thermochemical process (Li et al. 2022; Wang et al. 2020). The conventional agricultural application of this by-product is the improvement of the physical, chemical, and biological properties of the soil (El-naggar et al. 2019; Montanarella and Lugato 2013). However, novel alternative uses of biochar are currently under study, such as the adsorption of pollutants in soil and water (Oleszczuk and Zielin 2015; Catizzone et al. 2021; Gao et al. 2022) or the development of biochar-based catalysts for industrial reactions. However, their potential use depends on the physicochemical properties of the biochar. The primary advantage of biochar lies in its low cost, eco-friendly, and easy production, accompanied by a wide range of potential applications. This material also exhibits a significantly reduced environmental impact compared to alternative chemical-derived materials (Li et al. 2024).

Physical activation is typically carried out by exposing the biochar to high temperatures in the presence of activating gases, whereas chemical activation is applied by treating the biochar with chemical agents. Moreover, some activation processes for example those based on CO_2_ enhance the textural and superficial properties of biochar as an adsorbent material. In this sense, the properties of biochar are not only improved by increasing its surface area and introducing more functional groups and improve selectivity towards chemical groups, such as aromatic rings. This improvement is due to more available adsorption sites and better contact between the adsorbent and adsorbate (Zhang et al. 2019; Sevilla et al. 2021).

There is a lack of studies in the literature that investigate antibiotic adsorption on biochar in real aquaculture wastewater, since it is a complex matrix. Most available works use simulated wastewater or ultrapure solutions to investigate the performance and mechanisms of biochar adsorption. However, in this study, authors aim to develop sustainable and cost-effective treatment technologies for aquaculture wastewater treatment in real scenarios. More precisely, we focus on the preparation and characterization of activated biochars derived from pine bark, with the aim of establishing the most suitable process for biochar activation for antibiotic adsorption. Furthermore, this contribution assesses the interaction mechanisms between tetracycline and biochar, as well as the influence of the operational parameters to support the design and optimization of biochar-based treatment systems. In addition, we have studied the integration of biochar adsorption with AOPs, which represents a promising approach to enhance the removal efficiency of antibiotics.

## Materials and methods

### Biochar preparation and characterization

The carbon derived from the pyrolysis of pine at 450 °C was activated at 800 °C (at a rate of 10 °C/min) and maintaining the sample at this temperature for one hour. Activation was carried out under CO_2_ (BC_CO_2_) or Ar-steam (BC_H_2_O) under the previously detailed conditions. The resulting biochar samples were characterized using various techniques: X-ray diffraction (XRD), Scanning electron microscopy (SEM), N_2_ adsorption (BET analysis) and Fourier Transform Infrared Spectroscopy (FTIR).

Specific surface area was calculated from N_2_ adsorption isotherms at − 196 °C and CO_2_ adsorption data at 0 °C. Negligible changes were observed when the carrier gas was changed to CO_2._ X-ray diffraction (XRD) analysis was performed using a Philips X’pert Pro diffractometer equipped with a q/2q goniometer, CuKα radiation (λ = 1.540530 Å), and an X’Celerator detector. Fourier-transform infrared (FTIR) spectroscopy was conducted in diffuse reflectance mode (DRIFT) on a JASCO FT/IR-6200 IRT-5000 spectrometer. Physisorption isotherms were measured using a Micromeritics ASAP2010 analyzer. Surface morphology and elemental composition were investigated with a Hitachi S4800 SEM-FEG scanning electron microscope equipped with a Bruker-X Flash-4010 energy-dispersive X-ray (EDX) detector. Apart from the porous structure and the functional groups in the biochar, other parameters, such as the pH of zero charge (pH_PZC_) were determined to assess the influence of pH on tetracycline adsorption (Nidheesh et al. 2021).

### Chemicals

Potassium Peroxymonosulfate (PMS) was supplied by Oxone^®^. Tetracycline was supplied by Sigma-Aldrich with a purity of > 95% (wt./wt.). Acetonitrile (HPLC grade) and Acetic Acid (HPLC grade) were supplied by Merk^®^.

### Antibiotic quantification

Tetracycline concentration was determined via HPLC. The initial tetracycline concentration was 10 mg/l. An Agilent 1260 Infinity II LC System HPLC/VWD was employed, with a ZORBAX Eclipse Plus C18 (4.6 × 100 mm, 5 µm) column as a stationary phase and 2% acetic acid in water/acetonitrile 90:10 (vol.) at 1.5 ml/min as a mobile phase, without applying temperature control. A sample volume of 5 μL were injected into the system, and the total analysis time was 5 min. The absorption wavelength was fixed at 254 nm. Tetracycline concentration determinations were carried out in triplicate.

### Batch adsorption and PMS activation experiments

All adsorption and PMS activation experiments were conducted in 100 ml borosilicate glass batch reactors fitted with PTFE-lined caps to prevent evaporation and external contamination. Unless otherwise stated, the working volume in each experiment was 50.0 ml. The experiments were conducted under controlled laboratory conditions at 25 ± 1 °C with agitation atapproximately150 rpm to ensure homogeneous suspension of the biochar particles. Batch adsorption experiments were conducted for contact times between 1 and 240 min to obtain kinetic profiles, while equilibrium experiments were run for 24 h. At predetermined time intervals (1, 5, 10, 20, 30, 60, and 180 min), 2.0-ml aliquots were withdrawn using sterile syringes and immediately filtered through 0.22 µm PTFE syringe filters to remove suspended biochar prior to HPLC analysis.

For PMS activation experiments, the same procedure was followed with the addition of PMS to a final concentration of 0.01 mM after the initial adsorption stabilization period (30 min). PMS was freshly prepared before each experiment to avoid degradation. Control tests were included in triplicate: (i) PMS only (no biochar), (ii) Biochar only (no PMS), and (iii) Matrix blank. These controls allowed isolating adsorption, oxidation, and matrix-interference contributions.

#### Preparation of biochar suspensions

For each experiment, the desired mass of biochar (100–400 mg/l) was weighed using a precision microbalance (± 0.1 mg) and dispersed in ultrapure water or aquaculture wastewater. The suspensions were pre-conditioned for ca. 30 min before tetracycline addition to allow wetting and initial stabilization of biochar surface charges. Biochar particle size was < 250 µm to minimize internal diffusion limitations. Adsorption tests were initiated by adding tetracycline to a final concentration of 10 mg/l. The initial pH was adjusted (when required) using 0.1 M HCl or 0.1 M NaOH and verified using a calibrated pH meter (Crison GLP 22).

#### Experiments using real aquaculture wastewater

Aquaculture wastewater was collected from a recirculating aquaculture system (Universidad Politécnica de Madrid) and stored at 4 °C. All experiments using this matrix were performed within 48–72 h of collection. No dilution or filtration was applied to reproduce strict real conditions.

Aquaculture wastewater physicochemical characterization is summarized in Table 1, showing notable parameters such as high sulfate concentration (85.10 mg/l), elevated chloride levels (34.10 mg/l), and significant nitrate content (24.77 mg/l). The matrix also exhibited moderate turbidity (8.1 NTU) and alkaline pH (8.2), with a conductivity of 432.3 µS/cm, indicating a substantial presence of dissolved inorganic species.

**Table 1:** Characterization of aquaculture wastewater used in this study

Parameter	Value	Units
pH	8.2	
Conductivity	432.3	μS/cm
Turbidity	8.1	NTU
Ammonia	0.1	mg/l
Fluoride	0.16 ± 0.04	mg/l
Acetate	-	mg/l
Chloride	34.10 ± 0.06	mg/l
Chlorate	0.34 ± 0.01	mg/l
Nitrate	24.77 ± 0.04	mg/l
Nitrite	-	mg/l
Sulfate	85.10 ± 0.02	mg/l

#### Experimental reproducibility and data analysis

All experiments were performed in triplicate (*n* = 3). Results are expressed as mean ± standard deviation (SD). Adsorption capacity (qt) and removal efficiency (%) were calculated using standard mass-balance equations. Kinetic (pseudo-first-order, pseudo-second-order, intraparticle diffusion) and isotherm (Langmuir, Freundlich) models were fitted using OriginPro 2023, and goodness of fit was assessed via R^2^, RMSE error functions.

## Results and discussion

### Activation agent influence on biochar properties

First, regarding the influence of activation agents, it has been observed how activation with CO_2_ leads to a biochar with a large specific surface area and with micropores and mesopores, similar to powered activated carbon (Yue et al. 2014; Mogolodi Dimpe and Nomngongo 2019). While activation with humid Ar leads to a solid with a lower specific surface area (although still high) and the generation of macropores, as can be observed in Table 2.

**Table 2:** Textural properties of samples activated under different conditions

Sample	SBET (m^2^/g)	Total Pore Volume (cm^3^/g)	Micropore Volume (cm^3^/g)	Mesopore Volume (cm^3^/g)	Macropore Volume (cm^3^/g)
BC_CO_2_	583	0.455	0.145	0.326	-
BC_H_2_O	385	0.188	0.158	0.025	0.004

Secondly, regarding the textural properties, the results show that CO_2_ activated biochar (BC_CO_2_) exhibits a larger surface area and a better-developed porous structure than humid Ar activated biochar (BC_H_2_O). A strong influence of the activation agent in the development of surface area and microporous area has been reported in the literature (Huang et al. 2009; Tan et al. 2014). Other research has reported specific surface areas of 430 m^2^/g for similar agricultural residues such as cassava, 352 m^2^/g for sugarcane bagasse or 252 m^2^/g for potato leaves (Singh et al. 2020; Peñafiel et al. 2021).

**Fig. 1:**
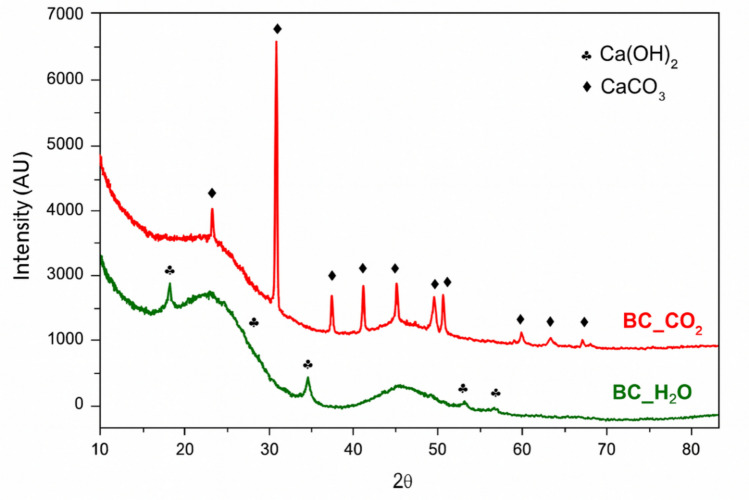
X-ray Diffraction (XRD) profiles of BC_CO_2_ and BC_H_2_O

According to the diffractograms, which are shown in Fig. 1, in the case of the sample BC_CO_2_, characteristic peaks of CaCO_3_ (01–085–1108) are detected, while in BC_H_2_O Ca(OH)_2_ is detected.

The decomposition temperature of CaCO_3_ is 825 °C, but in the presence of CO_2_ it is hindered. This is the most likely reason to explain why it is only detected in the case of the sample activated in CO_2_ (BC_CO_2_). The detection of Ca(OH)_2_ can be explained according to Eq. (1).

1 $$\mathrm{Ca}{(\mathrm{OH})}_{2} +\text{ C}{\mathrm{O}}_{2} \rightleftharpoons \text{ CaC}{\mathrm{O}}_{3} + {\mathrm{H}}_{2}\mathrm{O}$$

Furthermore, broad peaks detected between 18–28 and 42–47 degrees 2θ are associated to carbon presence. More precisely, the first of these can be attributed to the (002) crystallographic plane and is related to the degree of orientation of the aromatic sheets formed during pyrolysis, so that the sharper this peak, the greater the degree of laminar orientation. Moreover, the second peak corresponds to the (101) crystallographic plane and it is related to the size of the aromatic sheet (Li et al. 2016). The sharper the peak, the greater the degree of condensation of the aromatics. For these samples, the second of these peaks is similar in both samples, while the first is slightly smaller in the sample activated in CO_2_, so we can say that this sample has a higher degree of graphitization.

**Fig. 2:**
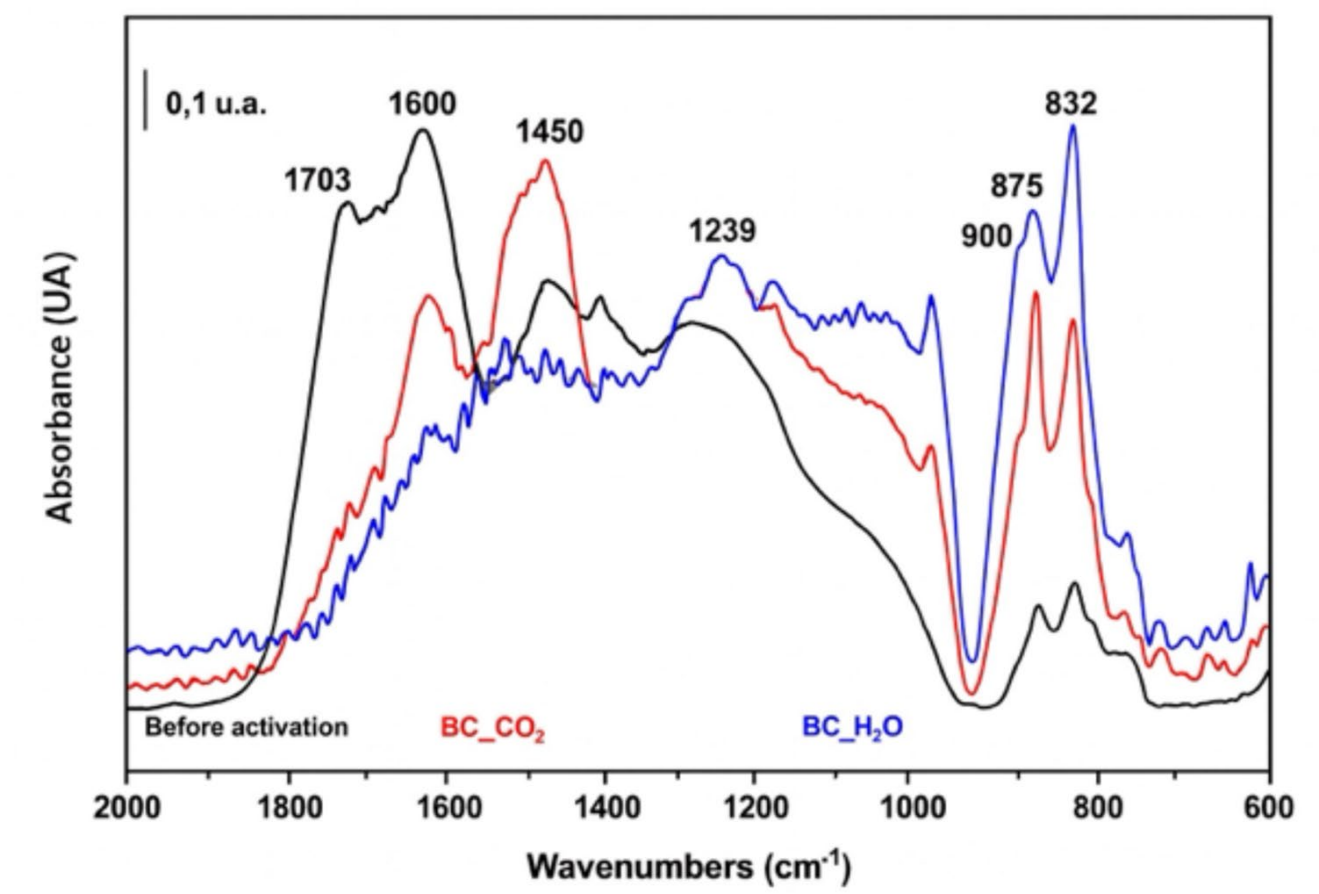
FTIR spectra of the sample prior to activation and activated in CO_2_ (BC_CO_2_) and in humid Ar (BC_H_2_O)

Characterization by FTIR spectroscopy shows the variations that occur during activation. Results are shown in Fig. 2, in which we can distinguish bands in 1703, 1600, 1450, 1239, 900, 875, and 832 cm^−1^.

The band at 1703 cm⁻^1^ indicates the presence of oxygenated compounds (aldehydes, ketones) due to C = O vibrations. This band, quite intense in the inactivated sample, almost disappears after the treatment at 800 °C, indicating that these functional groups are lost due to the influence of temperature. The C = C vibrations in aromatic compounds are detected around 1600 cm⁻^1^ while the = C-H vibrations out of the plane of these compounds appear in the region 900–700 cm⁻^1^. According to literature data, the intensity ratio (areas) of these bands allows estimating the degree of condensation of polyaromatic hydrocarbons. In our case, the intensity of the bands in the 900–700 cm⁻^1^ region increases with activation whereas the band at 1600 cm⁻^1^ decreases, indicating an increase in the degree of condensation of aromatics during activation. However, in the sample activated in CO_2_, the presence of carbonates (which have a vibration in this region, ~ 1450 cm^−1^) hinders quantitative analysis.

**Fig. 3:**
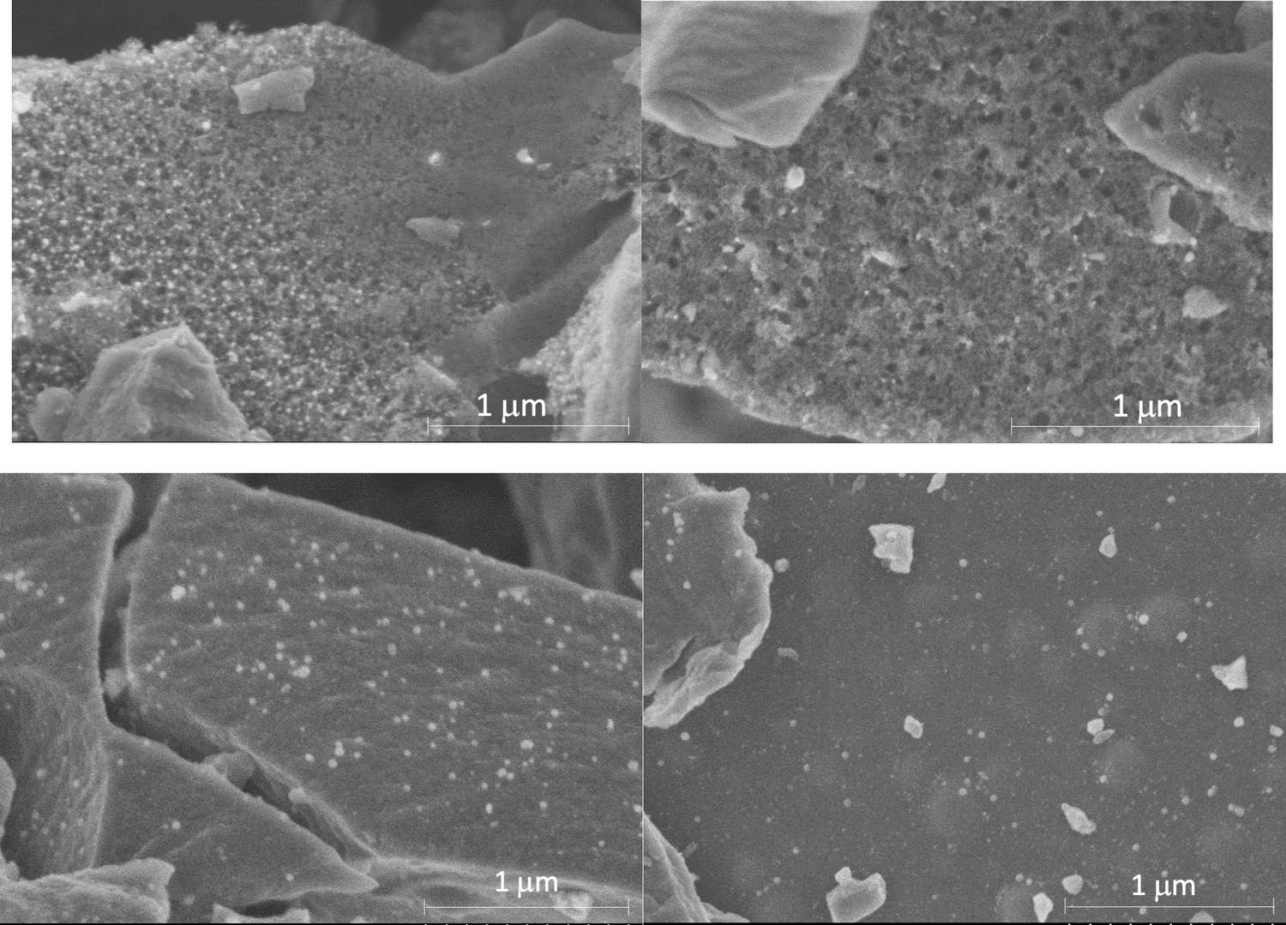
SEM images of the samples activated in CO_2_ (top) and humid Ar (bottom)

According to the SEM images (Fig. 3), the sample activated in CO_2_ is porous and contains CaCO_3_. In the case of the sample activated in humid Ar, fewer pores are detected, which is consistent with its lower specific surface area, and there are also whitish particles that are calcium compounds. In this case, some sort of ‘bubbles’ were observed under the surface that may be due to the formation of some species that diffuse outwards. The pH zero-point charge (pH_pzc_) was 6.7 for CO_2__BC and 7.4 for H_2_O_BC, respectively. This can be associated with the higher acidity of CO_2_ compared with humid-argon activation. In the literature, similar values are found for another CO_2_ activated biochars (Singh et al. 2020; Peñafiel et al. 2021; Gaye et al. 2025).

### Tetracycline adsorption in aquaculture wastewater

**Fig. 4:**
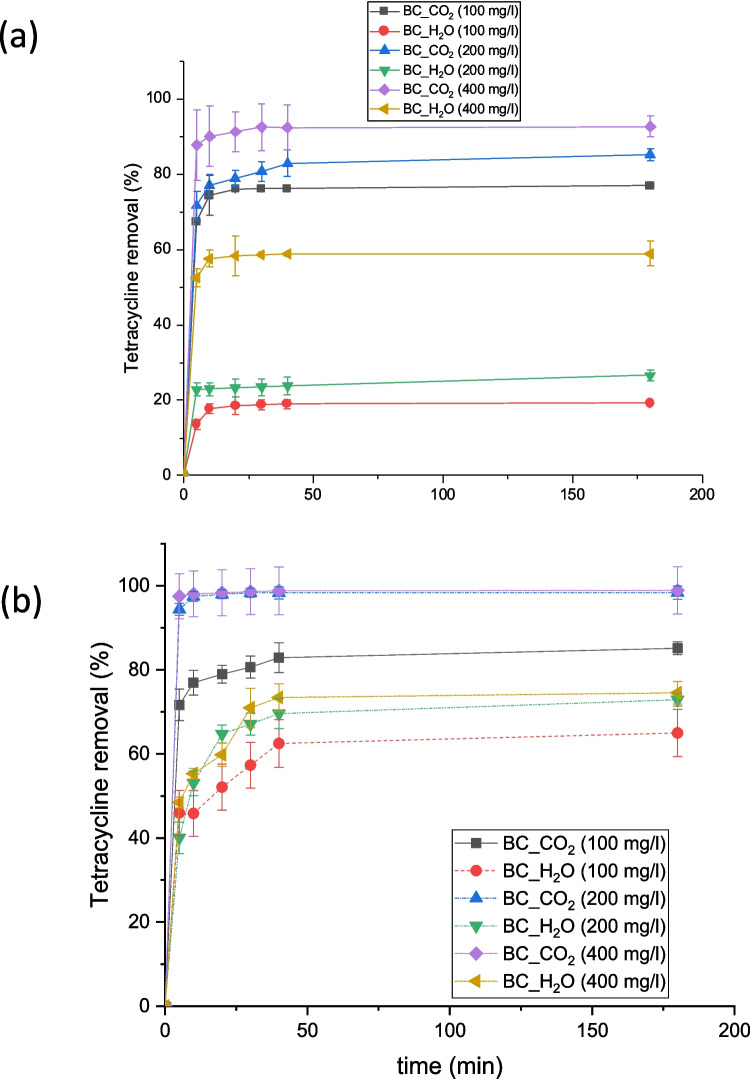
Tetracycline removal in (**a**) ultrapure water (UP) and (**b**) aquaculture water (AQ)

The removal efficiency of tetracycline from ultrapure (UP) and aquaculture water (AQ) was studied using the different biochar materials activated with CO_2_ (BC_CO_2_) or humid Ar (BC_H_2_O). The results are shown in Fig. 4.

Contrary to initial expectations, the removal efficiency, displayed in Fig. 4, was notably higher in the aquaculture water, particularly at lower biochar concentrations. This unexpected observation can be attributed to several interconnected factors, encompassing the physicochemical properties of the biochar, the water chemistry of the aquaculture environment, and the specific interactions between tetracycline and the biochar surfaces (Liping Zhang 2020).

Additionally, XRD patterns highlighted a higher degree of graphitization in the CO_2_-activated material. Fourier-transform infrared (FTIR) spectroscopy provided further insights into the chemical transformations occurring during activation, revealing the loss of oxygen-containing functional groups and an increase in aromatic condensation. The textural properties of the biochar also differed significantly. The CO_2_-activated biochar exhibited a larger specific surface area (583 m^2^/g) and a more extensive pore network, including a greater volume of micropores and mesopores, compared to the H_2_O-activated biochar (385 m^2^/g).

The unexpected superior performance of both biochars in aquaculture water can be explained by considering the unique characteristics of this water matrix. Aquaculture water typically exhibits higher ionic strength than ultrapure water, owing to the presence of dissolved salts such as sulfates (El Hanafi et al. 2024). This elevated ionic strength can enhance electrostatic interactions between the biochar surface and the tetracycline molecules, thereby promoting adsorption (Liping Zhang 2020). Hydrophobic interactions and complexation with organic matter could contribute to the overall removal efficiency (Kim et al. 2010). Therefore, the combined effects of higher ionic strength, higher pH, and the diverse constituents of aquaculture water create a more favorable environment for tetracycline adsorption, outweighing the inherent differences in surface area and pore structure between the two biochar materials. In the “Influence of pH” section, pH influence is studied to understand the overall adsorption mechanism. A stability test was performed to assess potential tetracycline release from the loaded biochars. No increase in antibiotic concentration was observed after 24 h in water, indicating negligible desorption and confirming the stability of the adsorbed species.

Regarding kinetic fitting, the results are shown in Table 3. The pseudo-second-order kinetic model provided the best fit for tetracycline adsorption onto biochar. This model implies that the adsorption rate is directly proportional to the square of the number of unoccupied adsorption sites on the biochar surface. Model performance was evaluated using the correlation coefficient (R^2^), the root means square error (RMSE) and the average relative error (ARE), to provide a more robust comparison between kinetic models.

**Table 3:** Kinetic adjust parameters in ultrapure water (UP)

Adsorbate	Kinetic Model	Parameters	R^2^	RMSE	% ARE
BC_H_2_O	Pseudo-first order	k_1_ = 0.0293 g/min	0.918	1.87	12.0
BC_H_2_O	Pseudo-second order	k_2_ = 0.0014 g/mg·min	0.996	0.42	2.4
BC_H_2_O	Elovich model	α = 5.72 mg/g·min, b = g/mg	0.982	0.61	6.1
BC_H_2_O	Intraparticle diffusion	k = 0.299 mg/(g·min^1/2^)	0.970	0.74	8.4
		I = 0.418 mg/g			
BC_CO_2_	Pseudo-first order	k_1_ = 0.0142 g/min	0.941	1.43	10.3
BC_CO_2_	Pseudo-second order	k_2_ = 0.030 g/mg·min	0.998	0.31	1.9
BC_CO_2_	Elovich model	α = 12.84 mg/g·min, b = g/mg	0.987	0.47	4.9
BC_CO_2_	Intraparticle diffusion	k = 4.86 mg/(g·min^1/2^)	0.808	2.28	15.8
		I = 22.31 mg/g			

The adsorption performance of the biochars developed in this study was evaluated using several kinetic models and supported by multiple statistical indicators (R^2^, RMSE, and ARE). For the BC_H_2_O sample, tetracycline adsorption followed a pseudo-second order (PSO) model with an excellent fit (R^2^ = 0.996, RMSE = 0.42, ARE = 2.4%), confirming that chemisorption is the dominant mechanism. The intraparticle diffusion (IPD) model also showed a relatively good fit (R^2^ = 0.970, RMSE = 0.74, ARE = 8.4%), suggesting that pore diffusion contributes to, but does not control, the overall adsorption rate. In contrast, the BC_CO_2_ sample exhibited an even stronger agreement with the PSO model (R^2^ = 0.998, RMSE = 0.31, ARE = 1.9%), whereas the IPD model showed notably poorer performance (R^2^ = 0.808, RMSE = 2.28, ARE = 15.8%), indicating that diffusion limitations are less relevant and that surface adsorption processes dominate. These trends are consistent with the kinetic constants obtained, k_2_ = 0.0014 g/(mg·min) for BC_H₂O and k_2_ = 0.030 g/(mg·min) for BC_CO_2_, reflecting the enhanced reactivity and availability of adsorption sites in the CO_2_-activated material (Yin et al. 2019; Berges et al. 2020).

These findings are consistent with previous studies reporting that tetracycline adsorption on biochars is primarily driven by specific interactions such as π-π bonding and electrostatic forces (Choi et al. 2008). For instance, biochars derived from agricultural waste exhibited adsorption capacities ranging from 2.98 to 8.23 mg/g, where π-π interactions were identified as the main adsorption mechanism (Huang et al. 2014). Similarly, sewage sludge-derived biochars activated with iron showed enhanced tetracycline adsorption due to increased surface area, porosity, and the presence of functional groups (Premarathna et al. 2019). Notably, wheat straw biochars demonstrated exceptionally high adsorption capacities, reaching up to 475.48 mg/g, although these values were strongly influenced by pH and temperature variations (Premarathna et al. 2019).

### Influence of pH

It is widely reported that at pH levels below the pH_pzc_ (Gaye et al. 2025; Premarathna et al. 2019), the positively charged biochar surface would attract the anionic tetracycline species, favoring adsorption. Conversely, at pH levels above the pH_pzc_, the negatively charged biochar surface repels the anionic tetracycline species, potentially reducing adsorption. The experimental results demonstrate an increase in tetracycline removal as pH increases, which aligns with the hypothesis that electrostatic interactions play a crucial role in the adsorption process. At pH = 9, both biochars exhibit the highest tetracycline removal, suggesting that the anionic form of tetracycline is the predominant species adsorbed under these conditions.

The experimental results show higher tetracycline (TC) removal at pH 9, even though both the biochar surface and TC molecules are negatively charged at this pH, which would normally result in electrostatic repulsion.

**Fig. 5:**
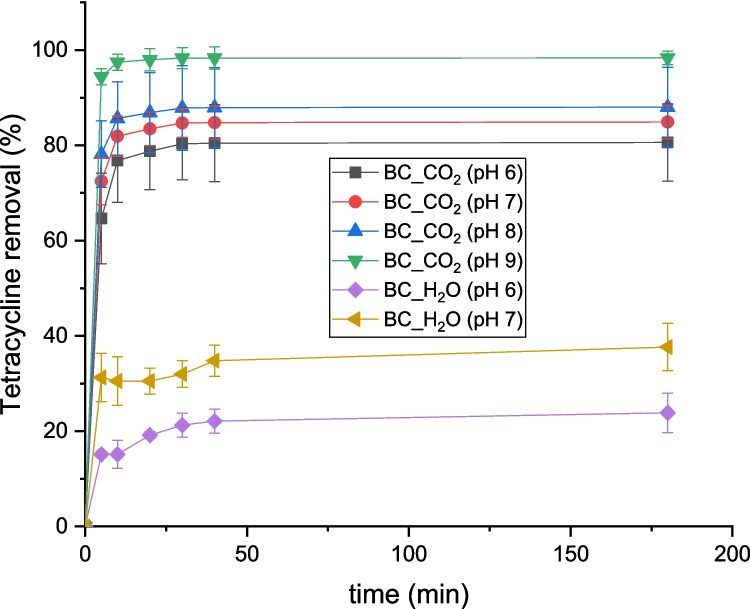
pH dependence tetracycline removal

As illustrated in Fig. 5, both BC_CO_2_ and BC_H_2_O performed better at pH 9, with the CO₂-activated biochar consistently achieving higher removal. The pH_pzc_ values of the biochars (6.7 for CO₂_BC and for H₂O_BC) indicate that at pH 9, their surfaces are negatively charged. At pH 9, tetracycline (TC) predominantly exists in its anionic form, having undergone deprotonation of key functional groups according to its three characteristic pK_a_ values: (i) pK_a_ = 3.3, corresponding to the loss of a proton from the tricarbonyl system; (ii) pK_a_ = 7.7, associated with deprotonation of the dimethylammonium group; and (iii) pK_a_ = 9.7, related to the deprotonation of the phenolic hydroxyl. As a result, TC carries one or two negative charges at pH 9, while the biochar surface, being above its point of zero charge, is also negatively charged. Despite this electrostatic repulsion, experimental results show enhanced tetracycline removal at pH 9, suggesting that non-electrostatic interactions such as hydrogen bonding, π–π stacking, surface complexation, and increased molecular diffusion into biochar pores become dominant mechanisms at alkaline pH, overriding the repulsive forces and enabling efficient adsorption (Berges et al. 2020). Results presented in this study are consistent with findings from another research. For instance, several authors have reported that the adsorption of tetracycline on biochar increases at higher pH levels. Berges et al. (2020) investigated the adsorption of four antibiotics (amoxicillin, enrofloxacin, sulfadiazine, and trimethoprim) onto activated carbon, with a focus on the influence of pH (Sajjadi et al. 2019). The study revealed that the adsorption of sulfadiazine and amoxicillin, which have pK_a_ values of 6.4 and 3.2, respectively, was enhanced in acidic conditions (pH < 7), while trimethoprim adsorption, with a pK_a_ of 7.1, was favored in basic conditions (pH > 7). Enrofloxacin adsorption, with a pK_a_ of 6.2, remained relatively unaffected by pH changes. The pH at the point of zero charge (pH_pzc_) of the activated carbon was determined to be 7.3. At pH levels below the pH_pzc_, the surface of the activated carbon is positively charged, while at pH levels above the pH_pzc_, it is negatively charged. The varying responses of the antibiotics to pH were explained by their differing chemical properties and ionization states at different pH levels (Liu et al. 2017).

### Biochar combined with PMS

**Fig. 6:**
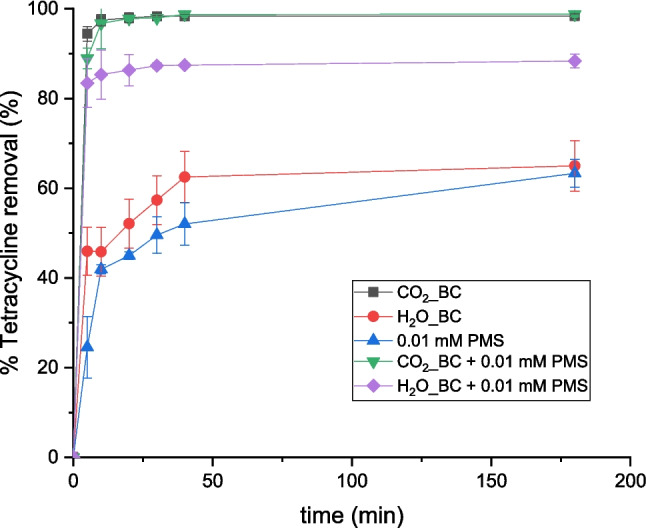
Tetracycline removal with PMS and biochar in aquaculture water (AQ)

The combination of biochar adsorption with peroxymonosulfate (PMS) activation represents an effective strategy, leveraging both adsorption capacity and advanced oxidation mechanisms. The results of this combination in real aquaculture water (AQ) are shown in Fig. 6.

In alignment with these findings, the results shown in Fig. 6 confirm that the synergistic application of PMS with both biochars significantly enhances tetracycline removal compared to adsorption alone. Moreover, the observed enhancement in removal efficiency upon PMS addition underscores the catalytic role of biochar in PMS activation, facilitating the generation of reactive species responsible for tetracycline degradation (Ma et al. 2020; Rodríguez-Chueca et al. 2020). According to the literature, biochar's surface functional groups and structural defects play a pivotal role in PMS activation and pollutant degradation (vom Eyser et al. 2015). FTIR analysis indicated notable differences in surface chemistry between both activation methods. The disappearance of the band at 1703 cm⁻^1^ after activation reflects the thermal decomposition of oxygenated functional groups (aldehydes, ketones), reducing hydrophilic interactions but potentially enhancing electron transfer processes involved in PMS activation. Simultaneously, the increased intensity of bands in the 900–700 cm^−1^ region points to a higher degree of aromatic condensation in the activated samples (see the “Biochar preparation and characterization” section). This structural evolution towards a more condensed aromatic matrix is particularly relevant, as sp^2^-hybridized carbon structures and π-electron systems facilitate PMS activation through electron transfer mechanisms. However, in BC_CO_2_ biochar, the presence of carbonates complicates quantitative FTIR analysis in this region, though their catalytic contribution via alkaline surface sites cannot be ruled out. Overall, these structural and chemical modifications enhance the biochar’s dual role as adsorbent and PMS activator, reinforcing the synergistic effect observed in tetracycline removal.

Characterization results revealed that BC_CO_2_ exhibits a larger specific surface area, and a more developed porous structure compared to BC_H_2_O. Such textural differences are crucial, as they increase the availability of active sites for PMS activation and pollutant adsorption, enhancing mass transfer and catalytic efficiency (Guerra-Rodríguez et al. 2018). The interaction between tetracycline molecules adsorbed onto the biochar surface and the generated radicals ensures a high probability of reaction, thus accelerating the overall degradation process (Gaye et al. 2025). Moreover, biochar acts as a catalyst to activate PMS, enhancing the generation of reactive radicals such as sulfate radicals and hydroxyl radicals. These radicals are highly oxidative and can readily degrade tetracycline. Finally, the adsorption of tetracycline onto the biochar surface places it close to the generated radicals. This proximity significantly enhances the probability of reaction between the radicals and tetracycline, leading to accelerated degradation. The surface functional groups on biochar may also participate in these reactions, further contributing to the synergistic effect.

Gaye et al. (2025) demonstrated this approach by synthesizing biochar from *Guiera senegalensis* waste and using it for the degradation of amoxicillin (AMX). Their study revealed that the synergistic effect of adsorption and PMS activation achieved AMX removal efficiencies exceeding 90% in various water matrices, despite the presence of inhibitory inorganic ions such as sulfates and phosphates. Comparing the removal efficiency of tetracycline in aquaculture water achieved in this study with other biochar-based PMS activation systems highlights the effectiveness of this approach, even under complex matrix conditions. In this work, the combination of adsorption and PMS activation achieved a removal efficiency of 99% using a PMS concentration of only 0.01 mM, outperforming many studies that required higher oxidant dosages or metal doping strategies. For instance, cobalt-impregnated biochar derived from spent coffee grounds reached 97% tetracycline removal but required 0.6 mM PMS (Nguyen et al. 2019). Similarly, Co- or Fe-supported composites achieved complete removal of contaminants such as sulfapyridine, chloramphenicols, and sulfamethoxazole, but with PMS dosages ranging from 1 to 10 mM (Li et al. 2020; Xu et al. 2020; Wang and Wang 2019). Although effective, these systems involve higher chemical inputs and the risk of metal leaching.

In contrast, the metal-free biochar used in this study offers a more sustainable and environmentally benign alternative, achieving comparable or superior performance with minimal PMS dosage. Other studies on the removal of emerging contaminants like bisphenol A, triclosan, and ofloxacin also reported high efficiencies (> 90%), but similarly relied on embedded metal catalysts or higher oxidant concentrations (Tang et al. 2018). Even simple biochar systems, such as rice husk biochar for tetracycline removal, required 20 mM PMS to achieve 90% removal (Huong et al. 2020), which is substantially higher than the conditions applied here. This underscores the enhanced catalytic potential of the biochar developed in this work, likely due to its optimized surface area, aromatic condensation, and functional group distribution.

## Conclusions

This study demonstrates that the activation agent plays a decisive role in tailoring the physicochemical properties of pine-bark biochars and, consequently, their effectiveness in removing tetracycline from aquaculture wastewater. CO_2_ activation produced biochar with a substantially larger surface area (583 m^2^/g) and a more developed micro–mesoporous structure compared to humid Ar activation (385 m^2^/g), which directly enhanced adsorption performance. Batch experiments revealed high tetracycline removal efficiencies (80–100%) under real aquaculture conditions, confirming the robustness of the biochars in complex matrices containing competing ions and dissolved organic matter. Adsorption kinetics were best described by the pseudo-second-order model for both biochars, supported by the lowest RMSE (0.31–0.42) and ARE values (1.9–2.4%), indicating chemisorption as the dominant mechanism. Although intraparticle diffusion contributed to adsorption, its higher RMSE and ARE values, particularly for BC_CO_2_, showed that diffusion was not the rate-controlling step. The adsorption process was strongly influenced by pH, with maximum removal observed under alkaline conditions (pH 9). Despite electrostatic repulsion expected above the pH_PZC_, enhanced removal suggests that non-electrostatic mechanisms, including π–π interactions, hydrogen bonding, and surface complexation are predominant at high pH. These findings are consistent with previous reports on antibiotic–biochar interactions and support the mechanistic interpretation developed in this work.

Another goal of this study was the integration of biochar adsorption with peroxymonosulfate (PMS). The combination produced a clear synergistic effect, achieving up to 99% removal at PMS dosages as low as 0.01 mM, substantially lower than many PMS-based systems reported in the literature, which often require ≥ 0.5–1 mM or metal-doped catalysts. This demonstrates the catalytic capacity of metal-free biochars to activate PMS via surface functionalities and condensed aromatic domains, offering a cleaner and more sustainable alternative to metal-based activators. The dual action of adsorption and oxidation provides a promising pathway for developing scalable, low-chemical-input treatment strategies for emerging contaminants. Future work should focus on long-term stability, regeneration, and continuous-flow applications to consolidate the applicability of these materials in real aquaculture facilities.

## Data Availability

The associated data of this will be provided upon request.
